# Progression on Mechanism and Therapeutic Implications of Neddylation in Lung Cancer

**DOI:** 10.32604/or.2025.071940

**Published:** 2026-01-19

**Authors:** Jiayu Zou, Yajie Lu, Jiaqi Li, Zhaokai Zhou, Fu Peng, Pu Qiu, Hailin Tang, Cheng Peng

**Affiliations:** 1 State Key Laboratory of Southwestern Chinese Medicine Resources, School of Pharmacy, Chengdu University of Traditional Chinese Medicine, Chengdu, 611137, China; 2 Affiliated Hospital of Guangdong Medical University, Guangdong Medical University, Guangzhou, 511436, China; 3 Department of Pharmacology, Key Laboratory of Drug-Targeting and Drug Delivery System of the Education Ministry, Sichuan Engineering Laboratory for Plant-Sourced Drug and Sichuan Research Center for Drug Precision Industrial Technology, West China School of Pharmacy, Sichuan University, Chengdu, 610041, China; 4 Department of Urology, The Second Xiangya Hospital of Central South University, Changsha, 410011, China; 5 National Clinical Research Center for Metabolic Diseases, The Second Xiangya Hospital of Central South University, Changsha, 410011, China; 6 State Key Laboratory of Oncology in South China, Guangdong Provincial Clinical Research Center for Cancer, Sun Yat-Sen University Cancer Center, Guangzhou, 510060, China

**Keywords:** Neddylation, lung cancer, cancer growth, metastasis, drug resistance, drug candidates

## Abstract

Lung cancer is the most common but fatal malignant tumor worldwide. Patients with lung cancer experienced a relatively low 5-year overall survival rate, and issues such as metastasis and drug resistance remain prominent challenges in its clinical management. Neddylation, a novel type of post-translational modification, was overactivated in lung cancer and was closely associated with its occurrence, development, metastasis, and drug resistance. This review systematically summarizes the biological process of neddylation and deeply explores the latest research progress on how neddylation affects lung cancer cell proliferation, metastasis, and drug resistance mechanisms, with a focus on its regulation of key molecules such as Cullin-RING E3 ligases and the SCCRO family. Meanwhile, it concludes the current advances in potential therapeutic agents targeting neddylation-related targets, including small-molecule compounds (such as Pevonedistat) and natural extracts (such as arctigenin). Finally, the review prospectively evaluates the application potential and questions requiring further exploration of neddylation in lung cancer treatment. In conclusion, we aim to systematically summarize the biological process of neddylation, critically explore its roles in lung cancer proliferation, metastasis, and drug resistance, and evaluate the therapeutic potential of neddylation-targeting agents.

## 1 Introduction

**Figure 1:**
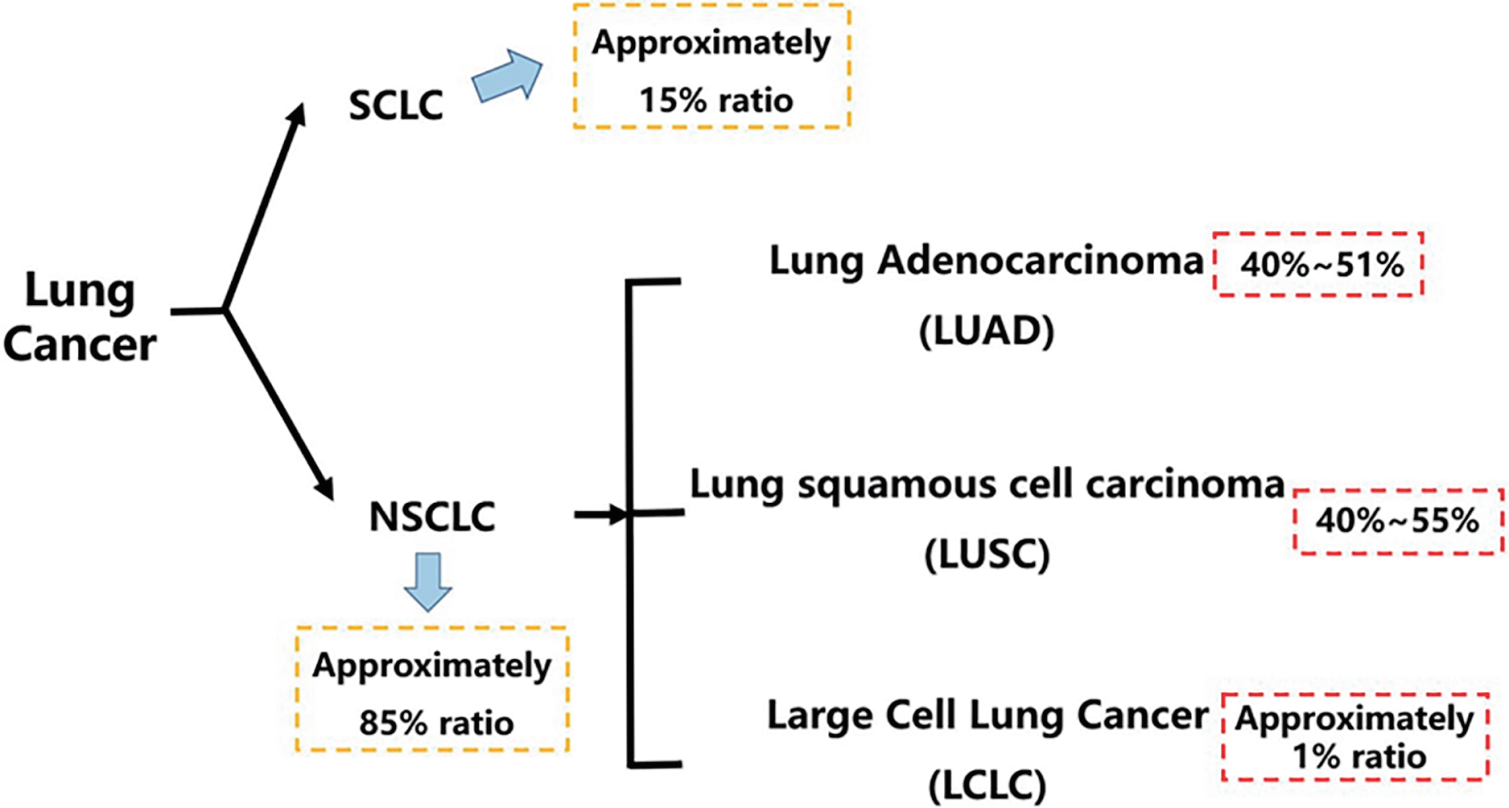
Histopathological classification of lung cancer. This figure outlines the histopathological classification of lung cancer, highlighting that NSCLC accounts for approximately 85% of all cases. It further breaks down NSCLC into its major subtypes, with LUAD and LUSC being the most prevalent. SCLC, small cell lung cancer; NSCLC, non-small cell lung cancer; LUAD, lung adenocarcinoma; LUSC, lung squamous cell carcinoma; LCLC, large cell lung carcinoma. Created in BioRender

Lung cancer is one of the leading malignant tumors in both morbidity and mortality in the world, which has posed a great threat to human health in the past decades [1,2]. According to histopathology, lung cancer is divided into small-cell lung cancer and non-small cell lung cancer [3] which accounts for about an 85% ratio as the main subtype, with a worse overall survival of patients in 5 years, and it is further classified into lung adenocarcinoma, large cell carcinoma, and lung squamous cell carcinoma, three subtypes [4,5] (Fig. 1). The classical strategies for the clinical treatment of lung cancer mainly include radiotherapy, chemotherapy, targeted therapy, and immunotherapy [6,7]. As a tumor escape process that can be regulated by various aspects, like the lung cancer microenvironment [8,9] and epithelial-mesenchymal transition [10]. The lung cancer metastasis contains the brain [11], lymphatics [12] and bone marrow [13] metastasis, etc., with its specific mechanism hasn’t been completely explored yet, significantly threatening clinical survival and prognosis of lung cancer patients. At the same time, lung cancer patients are also encountering drug resistance, like platinum resistance [14], epidermal growth factor receptor (EGFR)-TKI resistance [15], and PD-1 inhibitor resistance [16,17], etc.

**Figure 2:**
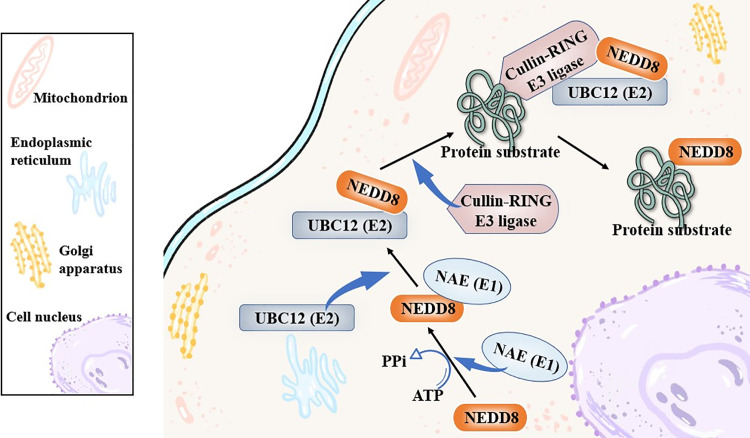
The canonical biological process of neddylation. The figure outlines the canonical neddylation cascade: NEDD8 is activated by E1 (NAE), transferred to E2 (like UBC12), and finally conjugated to substrate proteins (like Cullins) via E3 ligases (like CRLs), which modulates substrate activity. NEDD8, neural precursor cell expressed developmentally down-regulated protein 8; NAE, NEDD8-activating enzyme; UBC12, ubiquitin carrier protein 12. Created in BioRender

Neddylation as a new type of post-translational modification (PTM) was first initiated by NEDD8/Rub1, a small ubiquitin-like protein that could be activated via a combination with the NEDD8-activating enzyme (NAE, E1). Then, in this process, the activated NEDD8 would bind to the NEDD8-conjugating enzyme, such as ubiquitin carrier protein 12 (UBC12), ubiquitin-conjugating enzyme E2 F (UBE2F), ubiquitin-conjugating enzyme E2 M (UBE2M), E2, next, and the E2 cascaded catalyzed NEDD8 covalently linking to its protein substrates specifically in the end [18,19]. Normally, the linkage between NEDD8 and its protein substrates was achieved with a help of the Cullin-RING E3 ligase (CRL) [20], and the cullins family was the most common and canonical substrates of NEDD8 neddylation [21,22] (Fig. 2). Meanwhile, apart from cullin-RING E3 ligase protein substrates, there was other non-canonical protein substrates of NEDD8 that was identified. In recent years, various studies have revealed a high relevance between neddylation and the squamous cell carcinoma-related oncogene (SCCRO) family, which was also known as defective in cullin neddylation 1 domain containing 1 (DCUN1D1) or defective in cullin neddylation 1 (DCN1), and often, it would affect the cullin neddylation. In 2008, it was reported that the SCCRO (DCUN1D1) also served as a key component of the neddylation E3 complex, and it could participate in but hamper the assembly and activity between CAND1 and cullin-RING E3 ligase complex [23]. Later, researchers followed it and put effort into the specific role of SCCRO in neddylation, emphasizing its broader and pleiotropic effect in cullin neddylation [24]. Other Cul3-anchored complexes, KLHL9/KLHL13, were not affected by the SCCRO, highlighting the truth that the SCCRO was selectively regulating cullin neddylation *in vivo* [25].

As a burgeoning therapeutic target, neddylation exhibited significant potential in various types of cancers, presenting great study prospects. Through screening databases like PubMed, Web of Science, and Springer, etc., on neddylation in cancer treatment, in a criterion of the number of articles to sort, neddylation was more explored in lung cancer, liver cancer, breast cancer, prostate cancer, colorectal cancer, and stomach cancer [26–28]. As Lung cancer ranks as the top cause of cancer-related mortality all over the world [29], investigating the role of neddylation in lung cancer as a potential target is beneficial. This review aims to summarize the role of neddylation in lung cancer, focusing on its mechanisms in metastasis and drug resistance, while highlighting potential therapeutics targeting this pathway.

## 2 The Critical Role of Neddylation in Lung Cancer

### 2.1 Regulation of Neddylation Significantly Influencing Lung Cancer Growth

#### 2.1.1 Prognostic Value of Neddylation Patterns from Bioinformatic Studies

Through bioinformatic analysis, there was other evidence indicating the significant value of neddylation to lung cancer. Jabs’s group performed a genome-wide analysis of a NSCLC patient cohort (n = 190), among which ubiquitination and neddylation were frequently discovered in the highly correlating genes. Then, they conducted a meta-analysis of 1779 patients, finding an overlap between the survival-associated genes and highly correlating genes (61 to 301) like CAND1, a gene that was corroborated to participate in cancer metastasis [30,31].

A recent study focused on the neddylation-associated genes to explore their value in lung adenocarcinoma (LUAD). Researchers performed Univariate Cox analysis, identifying 76 neddylation-related prognostic genes first. Then, for the next step, an unsupervised consensus clustering analysis classified the experimental patients into two patterns. Then, several prognostic significant differences were found in which neddylation-cluster1 showed lower levels of immune infiltration than neddylation-cluster2, enhancing the tumor progression. They later performed the LASSO-Cox and multivariate stepwise regression approaches, and they successfully constructed a 10-gene prognostic signature. Finally, the results of neddylation-related risk score confirmed its value for the prediction of several clinical parameters like patient outcomes, chemotherapeutic responses, and gene mutation levels. From above, it indicated that assessment of the neddylation patterns had a large potential for telling the prognosis signatures of LUAD patients, which was available for a clinical personalized treatment design [32]. These findings highlight the potential of assessing neddylation patterns for prognostic stratification and personalized therapy in LUAD.

#### 2.1.2 Targeting NAE and NEDD8 Depletion to Suppress Lung Cancer Progression

It had been early proven that when blocking the NAE, a key factor of the neddylation process, the lung cancer progression would be significantly suppressed [33]. The NEDD8-depletion could suppress lung cancer progression owing to its induction of cullin-RING ligase substrates (p21, p27, and Wee1) accumulation, which was conform with the mechanism when using NAE-inhibitor to target the neddylation process. It would also arrest lung cancer cell cycle at the G2 phase and trigger cancer apoptosis or cell senescence later.

#### 2.1.3 Roles of Key Neddylation Enzymes: UBC12 and UBE2F

There were other studies specifically focusing on the effect of the NAE downstream NEDD8-conjugating enzyme, UBC12 (UBE2F/UBE2M), in lung cancer. The NEDD8 conjugating enzyme UBC12, as a target, has been discovered that it is also capable enough affecting the neddylation process for lung cancer treatment. They first detected the expression levels of UBC12 in the H1299 cell line through a western blot assay and performed bioinformatics analysis for estimation, observing the same variation as NAE-inhibition when UBC12 was knocked down. The results revealed that compared to normal lung tissues, UBC12 was highly expressed in lung cancer tissues and cells, and it showed a positive correlation. The UBC12-knockdown effectively hampered various phenotypes of lung cancer, especially arresting the cell cycle in the G2 phase. In the mechanism, the UBC12 knockdown also affected cullin neddylation, which later inactivated Cullin-RING E3 ligases and triggered tumor-suppressive CRL substrates accumulation, which finally inhibited malignant phenotypes like cell cycle [34].

By employing abundant experimental methods, it was discovered that the specific E3 (CRL5) and ubiquitin linkage (K11 Linkage) co-participated in the UBE2F neddylation process to promote lung cancer survival with a phorbol-12-myristate-13-acetate-induced protein 1 (NOXA, also known as PMAIP1) degradation. In this research, immunochemistry and immunoblotting assays were first used for the UBE2F protein expression variation detection. Then, a series of *in vitro* and *in vivo* conventional experiments were performed to confirm the anticancer bioactive value of targeting UBE2F. As for NOXA (PMAIP1), it was assessed by ubiquitylation assays, and the specific upstream participant of NOXA (PMAIP1) was also measured through a pulldown assay. Finally, the UBE2F was discovered to an overexpressed in NSCLC cells and patients, promoting lung cancer progression, but with a reversibility when it was silenced. Corresponding to it, the NOXA (PMAIP1) was also found to a downregulated and it could be reversed. These results elucidated that the UBE2F achieved its effect through inducing Cullin 5 neddylation, and it activated the CRL5 as an upstream molecular via K11 linkage to ubiquitylate NOXA (PMAIP1) for degradation, instead of K48 linkage. Besides, the validation of the CRL5-K11-NOXA axis was proved by a rescue assay, by which the NOXA knockdown was confirmed reversing the apoptosis induced by CRL5-silence or K11R mutation [35].

Moreover, interestingly, another NEDD8-conjugating enzyme, UBE2M, had a dual E2 effect on UBE2F. Normally, it could activate Cullin 3-Keap 1 E3 to promote UBE2F-mediated neddylation. However, UBE2M would also head for Parkin-DJ-1 E3 to degrade UBE2F when under stressed conditions. Then, it led to CRL5-inactivation together with its downstream NOXA-accumulation later, inhibiting lung cancer cell growth finally [36].

#### 2.1.4 Divergent Roles of the SCCRO Protein Family in Lung Cancer

**Figure 3:**
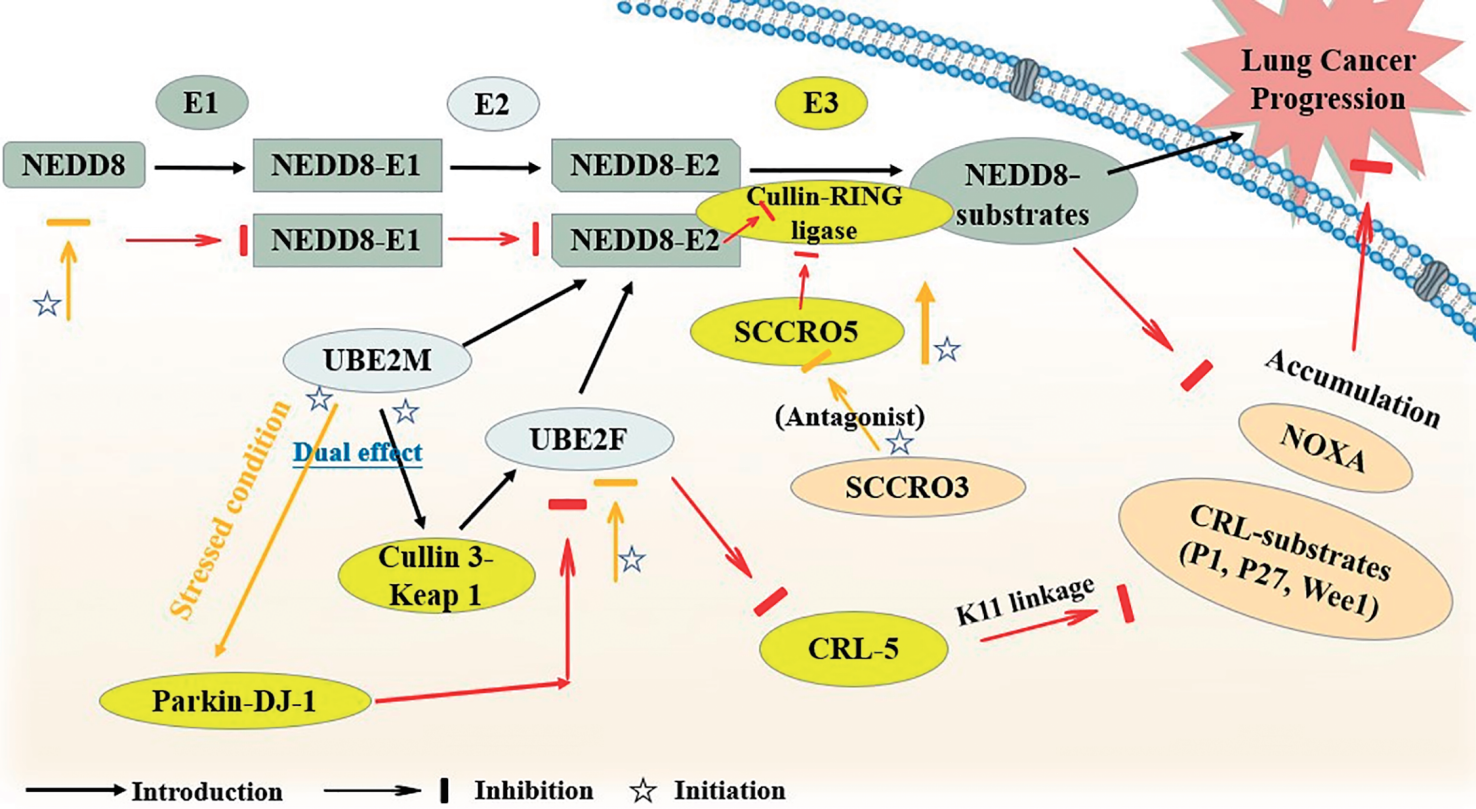
The brief mechanism of neddylation in lung cancer. The figure illustrates how SCCRO5 enhances cullin neddylation to promote CRL activity, whereas SCCRO3 antagonizes this process by blocking cullin nuclear translocation and lacking E3 activity, thereby exerting opposite effects on lung cancer progression. UBE2M, ubiquitin-conjugating enzyme E2 M; UBE2F, ubiquitin-conjugating enzyme E2 F; SCCRO, squamous cell carcinoma-related oncogene; CRL, cullin-ring ligase; NOXA (PMAIP1), phorbol-12-myristate-13-acetate-induced protein 1; Parkin-DJ-1, a functional complex between the E3 ubiquitin ligase parkin and the oncoprotein DJ-1. Created in BioRender

Apart from it, the neddylation-related SCCRO family was also discovered to have diverse bioactivities in lung cancer. A study confirmed an oncogenic potential of SCCRO5, serving as a neddylation E3 component in lung cancer, and SCCRO5 achieved its E3-like function mainly through an acceleration of cullin neddylation. First of all, researchers evaluated 203 randomly selected primary cancer tissue samples with their SCCRO5 mRNA and Protein levels detected by real-time PCR and Western blot assays. Other experiments conclude a knockdown of SCCRO5 by RNAi, and structure-function studies for the detection of SCCRO5 residues were also performed. Finally, the results revealed that SCCRO5 negatively regulated the lung cancer cell survival. The loss of SCCRO5 could lead to an oncogene addiction in lung cancer cells with a high endogenous level, and the lung cancer cell viability was selectively decreased [37]. However, SCCRO3, an SCCRO paralogue present in the human membrane, was proven to serve as an antagonist towards the neddylation activity of SCCRO and suppress the progression of lung cancer. The specific mechanism was on account of SCCRO3 working as a blocker of cullin nuclear translocation, thus it sequestered cullins to the membrane. Besides, unlike other SCCROs, SCCRO3 was neither capable enough to bind to UBC12 effectively, lacking the E3 activity [38] (Fig. 3).

In summary, key components of the neddylation pathway, such as UBC12, UBE2F, and SCCRO5, are frequently overexpressed in lung cancer tissues. Crucially, this aberrant overexpression is closely associated with poor patient prognosis. Multiple bioinformatic analyses and clinical sample validations have indicated that high expression levels of these genes predicted poor survival [34,37]. Therefore, utilizing the expression levels of these critical neddylation genes as biomarkers holds promise not only for predicting disease progression but also for developing targeted prognostic assessment tools.

### 2.2 Inhibition of Neddylation Significantly Suppressing Lung Cancer Metastasis

Focusing on the Cul-1 in 128 cases of human lung cancer samples and comparing with normal lung tissues, it revealed that 40% (51/128) of all samples showed Cul-1 overexpression, containing NSCLC, carcinoids, and high-grade neuroendocrine lung carcinomas. Besides, Cul-1 was found modified by neddylation in these samples, and the neddylated forms of Cul-1 later led to Cul-1 inhibitor CAND1 low-expression (*p* = 0.03) and cyclin E over-expression, implying the Cul-related neddylation pathways as a possible factor for highly aggressive lung cancer development [39].

**Figure 4:**
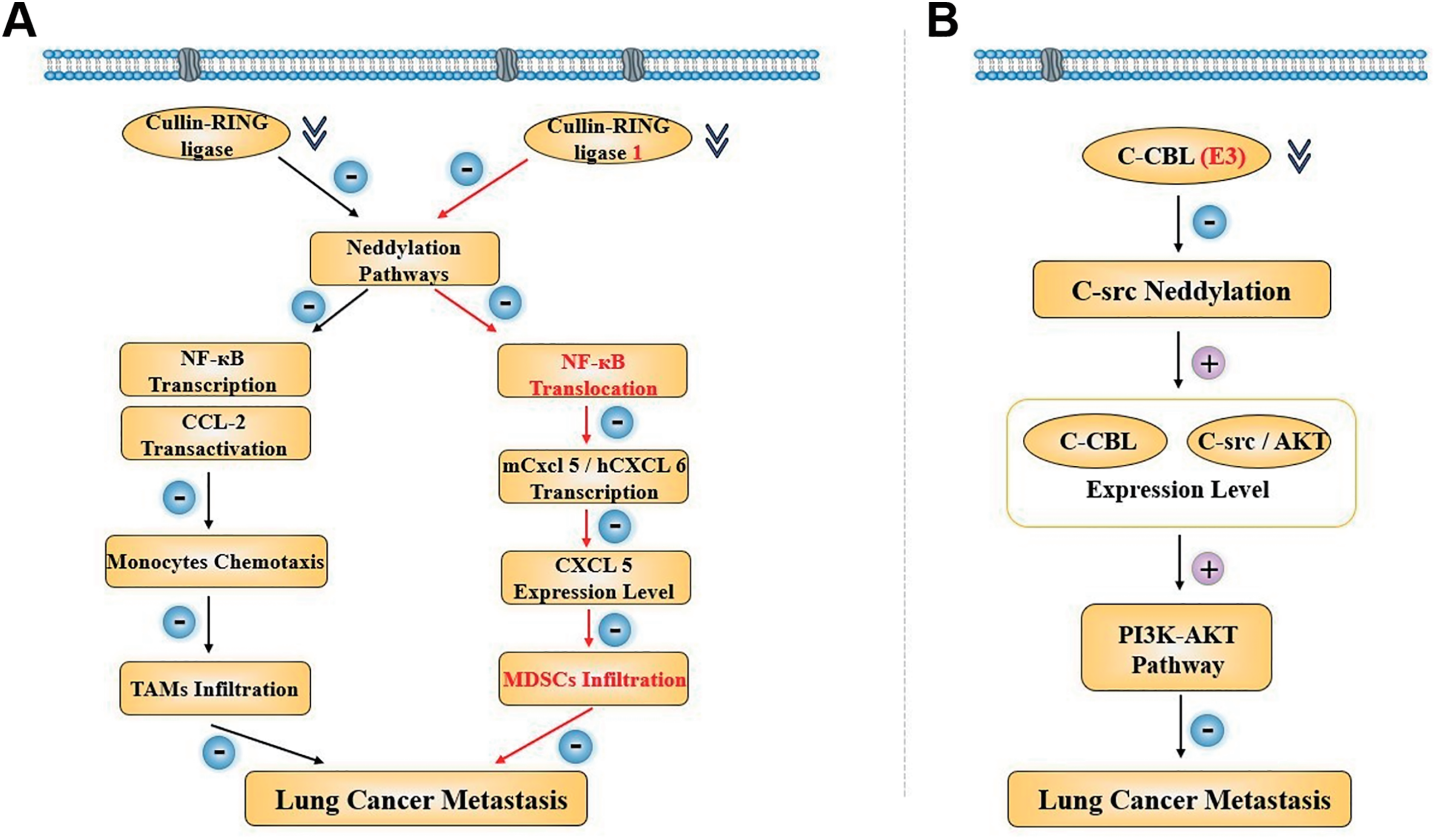
Two regulatory pathways concerning neddylation in lung cancer metastasis when the E3 was inhibited. (**A**) CRL-related neddylation pathway affecting two different immune cells’ infiltration to regulate lung cancer metastasis. (**B**) C-CBL serves as the E3 to regulate lung cancer metastasis through an effect on the c-src neddylation and PI3K-AKT pathway. NF-κB, nuclear factor kappa-light-chain-enhancer of activated B cells; CCL, c-c motif chemokine ligand; TAM, tumor-associated macrophage; CXCL, c-c motif chemokine ligand; MDSC, myeloid-derived suppressor cell; C-CBL, casitas b-lymphoma; AKT, AXL receptor tyrosine kinase; PI3K, phosphatidylinositol 3-kinase. Created in BioRender

Other evidence indicates that neddylation is involved in lung cancer metastasis. A study showed that neddylation did affect lung cancer migration, and it also confirmed a lower potential metastasis risk when neddylation inhibitors were used to block the neddylation process for clinical lung cancer treatment. Researchers were mainly centered on casitas b-lymphoma (C-CBL), an neddylation E3 ligase for proto-oncogene c-src, which was discovered both in human lung cancer cells and tissues. They found cell migration being stimulated by its upstream PI3K-AKT pathway when it was inhibited owing to the proto-oncogene c-src neddylation. In patients, also, the expression level of C-CBl and c-src/AKT was decreasing correspondingly, and the cancer metastasis was activated, leading to a poor survival [40]. Through an RNA-sequencing analysis, it was proven that the neddylation pathways would contribute to an inhibition of cancer metastasis through TAMs infiltration inhibition, along with a suppression of CCL2 transactivation and a lower NEDD8 expression level. Then, they found out that the CCL2 transactivation activity was affected by its upstream Cullin-RING ligases. At the same time, the NF-kappaB transcriptional activity was also silenced by it. Thus, by inhibiting cancer neddylation, lower expression level of CCL2 meant lower chemotaxis of monocytes, resulting in decreased TAMs infiltration, and consequently, suppressing lung cancer cell metastasis with better clinical overall survival. In 2022, a study also elucidated that neddylation was capable enough as a regulator for Myeloid-derived suppressor cells (MDSCs) infiltration in lung cancer. The NEDD8 was observed to be positively associated with MDSCs infiltration in LUADs, and the MDSCs infiltration could be suppressed when the related neddylation pathway was inactivated, together with murine CXCL5 expressing less. Through further exploration, the mechanism was similar to the TAMs that we had discussed above. The Cullin-RING ligase 1 was found to a downregulated, which later led to a blockage in the NF-kappaB translocation, with mCxcl5 or hCXCL6 transcriptional activity inhibited. It not only revealed an involvement of neddylation-NF-kappaB-m-CXCL5 axis in lung cancer MDSCs infiltration, but also showed the therapeutic potential of the neddylation process serving as a valuable treatment target for lung cancer clinical strategy [41] (Fig. 4).

### 2.3 Targeting Neddylation to Moderate Lung Cancer Drug Resistance

Studies were pointing to a connection between neddylation and drug resistance in lung cancer. In EGFR-mutant lung cancer, overexpression of GAS6 was observed both *in vitro* and *in vivo*. Subsequently, it was demonstrated that the overexpression of GAS6 was regulated by AXL receptor tyrosine kinase (AXL)—a receptor for GAS6—which correlated positively with GAS6 levels. Specifically, AXL modulated the T790M mutation, thereby contributing to the overcoming of EGFR inhibitor resistance [42]. Mechanistically, it was revealed that AXL exerted this effect primarily by promoting neddylation. As a trigger for low-fidelity DNA polymerases, AXL could facilitate neddylation to activate RAD18—the organizer of these polymerases—ultimately eliminating drug resistance [43].

Another study indicated a potential for targeting neddylation as a platinum-sensitization strategy for lung cancer clinical therapy. They first discovered that lung cancer cells could escape from platinum-induced apoptosis through an upregulation of the UBE2F [44]. Then, a mechanistic study showed that in platinum-resistant lung cancer cells, the UBE2F was weakly associated with the CRLs component Ring-box protein 1 (RBX1), and this was owing to the platinum, which hampered the formation of the UBE2F degradation complex, hence leading to UBE2F accumulation. Later, the neddylation of cullin5 was accelerated by the accumulated UBE2F, and the CRL5 substrate NOXA (PMAIP1) was downregulated consistently. Besides, they also performed a rescue assay, and the NOXA protein level was upregulated to induce lung cancer cell apoptosis when UBE2F was knocked out. It not only revealed the correlation between neddylation and lung cancer, but also emphasized the significant value of targeting neddylation for clinical chemotherapy-resistant lung cancer patients overcoming drug resistance [45].

## 3 Potential Drugs Targeting Neddylation for Lung Cancer Treatment

**Figure 5:**
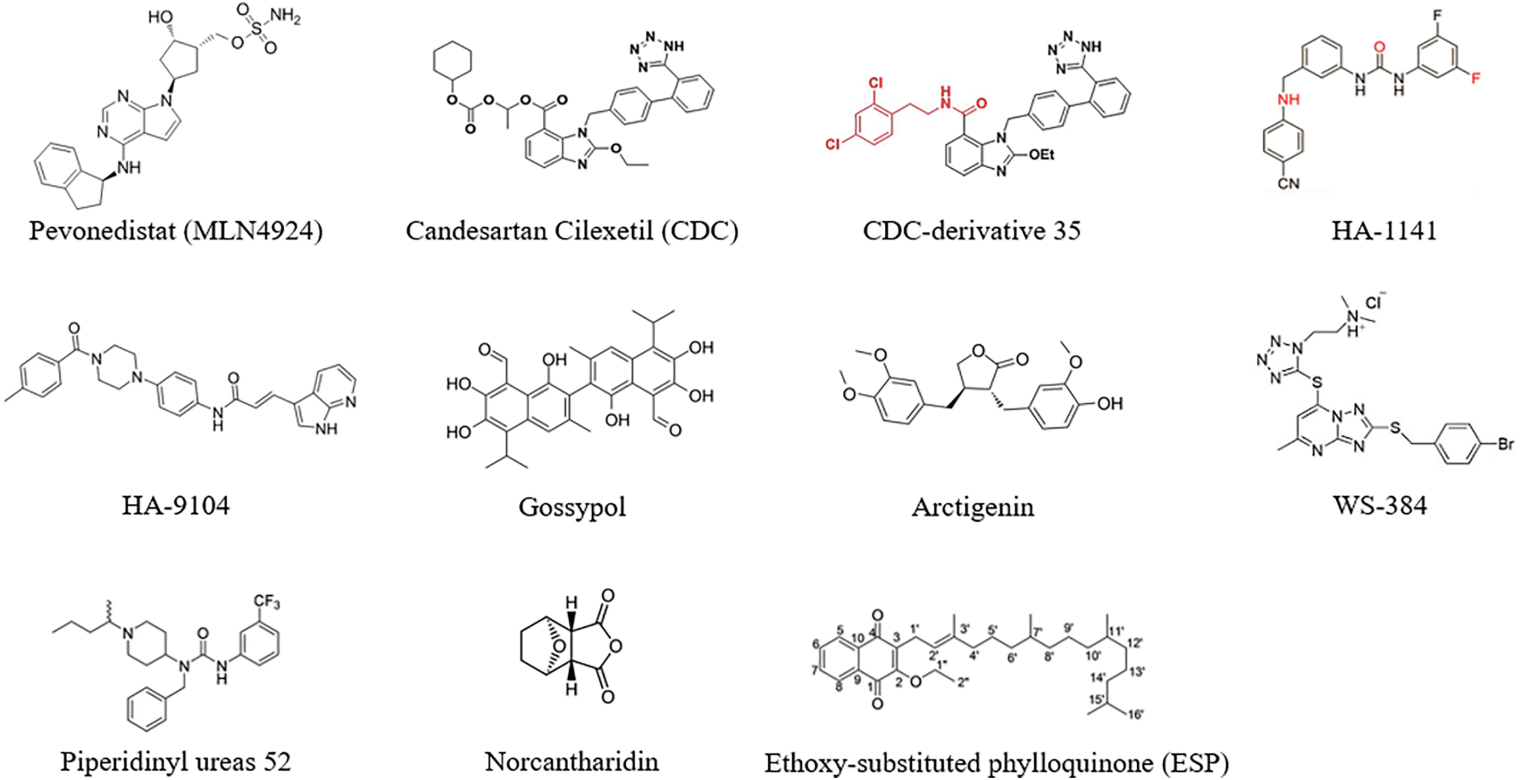
The molecular formula of potential drugs targeting neddylation. Created in ChemDraw

The potential compounds and drugs were capable enough to inhibit lung cancer progression through targeting neddylation and related pathways, with classification of their specific mechanisms summarized below (Table 1) (Fig. 5).

**Table 1:** Potential drugs targeting neddylation for lung cancer treatment

Category	Molecular/Drug	Regulation effect on neddylation in NSCLC	Brief mechanism	Function study	Reference
Small molecule compounds & chemical drugs	Pevonedistat (MLN4924)	↓	As NAE-inhibitor; Inactivation effect on Cullin-RING E3 ligases, leading to cell proliferation inhibition, apoptosis, cell cycle arrest, DNA damage and NOXA (PMAIP1) accumulation.	*In vitro* and *In vivo*	[46–50]
		↓	Inhibit MMP2, MMP9 and vimentin, etc. to affect lung cancer metastatic colonies’ intravascular survival, extravasation and formation.		[51]
		↓	Hamper the BRCA1 complex components’ recruiting to DNA damage sites, showing a synergistic effect with PARP inhibitors.		[52,53]
	Candesartan Cilexetil (CDC) and its derived compound	↓	Inhibited the cullin neddylation through competing with ATP to achieve the loss of NAE function.	*In vitro* and *In vivo*	[54,55]
	HA-1141	↓	Directly bind to NAE and inhibit the cullin 1-5 neddylation; Suppress lung cancer cell proliferation; Dual effect to induce autophagy.	*In vitro* and *In vivo*	[56]
	HA-9104	↓	Selectively target the UBE2F to inhibit the cullin-5 neddylation; Trigger apoptosis, cell cycle arrest and NOXA accumulation.	*In vitro* and *In vivo*	[57]
	WS-384	↓	Promoted expression of p21 by blocking cullin 1 neddylation and diminishing H3K4 demethylation at the CDKN1A promoter.	*In vitro* and *In vivo*	[58]
	Piperidinyl ureas	↓	Reduce the steady-state levels of neddylated CUL1 and CUL3; Hamper the DCN1-UBE2M interaction.	*In vitro*	[59]
	Ethoxy-substituted phylloquinone (ESP)	↓	Decrease neddylation pathway-related proteins expression; Affect the EGFR signaling and upregulate STAT1 and NDRG1 protein levels; Arrest the cell cycle at the G2-M phase.	*In vitro*	[60]
Natural & extracted drugs	Arctigenin	↓	Hamper the activity of UBC12 enzyme to affect the cullin neddylation and downstream pathways; Increase the tumor suppressor PDCD4 protein levels.	*In vitro*	[61]
	Gossypol	↓	Direct bind to SAG)-CUL5/RING-RBX1-CUL1 complex; Accumulation of CUL5, CUL1-substrates, MCL1, and NOXA (PMAIP1).	*In vitro*	[62]
	Norcantharidin	↑	Promote cullin 1 bind to CDC6; Mediate cullin 1-neddylation to promote CDC6 degradation.	*In vitro*	[63]

Note: Abbreviation: BRCA1, breast cancer susceptibility gene 1; CDC, candesartan cilexetil; CDKN1A, cyclin-dependent Kinase Inhibitor 1A; CUL, cullin; DCN, defective in cullin neddylation; EGFR, epidermal growth factor receptor; H3K4, histone H3 lysine 4; MMP, matrix metalloproteinases; NAE, NEDD8-activating enzyme; NDRG1, N-Myc downstream-regulated gene 1; NSCLC, non-small cell lung cancer; NOXA (PMAIP1), phorbol-12-myristate-13-acetate-induced protein 1; PARP, poly(ADP-ribose) polymerase; PDCD4, programmed cell death 4; RBX1, RING-box protein 1; SAG, sensitive to apoptosis gene; STAT1, signal transducer and activator of transcription 1; UBC12, ubiquitin carrier protein 12; UBE2F, ubiquitin-conjugating enzyme E2 F; UBE2M, ubiquitin-conjugating enzyme E2 M.

### 3.1 Small-Molecule Inhibitors for Molecularly Targeted Neddylation in Lung Cancer Therapies

As small-molecule inhibitors for neddylation could block the process of neddylation [64,65]. More attention could be paid to the potential as an alternative approach for lung cancer treatment.

#### 3.1.1 Pevonedistat (MLN4924)

Over the past few years, Pevonedistat (MLN4924) has been the most canonical NAE-inhibitor used for cancer research and exhibited prominent therapeutic value via targeting the neddylation process [46–49]. Previous studies had already proved that MLN4924 suppressed cancer progression due to its inactivation effect on CRL. Then, it led to an accumulation of CRL-substrates and NOXA (PMAIP1), which would induce cancer apoptosis consequently [33]. Another study explored the effect of MLN4924 in paclitaxel (PTX)-resistant lung cancer cells (PTX-resistant A549 and PTX-resistant H460) and successfully elucidated its therapeutic potential for clinical chemotherapy resistance. They found MLN4924 exerted its anti-resistance effect also through inactivating Cullin-RING E3 ligase and hampering the neddylation process, which subsequently not only led to an inhibition of cancer cells proliferation and spheroids formation, but also promoted apoptosis and DNA damage in them. However, even though the anti-PTX-resistance effect of MLN4924 was shown, no synergistic effect was shown when MLN4924 was cotreated with PTX in PTX-resistant NSCLC cells [50]. MLN4924 exerted prominent anticancer bioactivity through affecting the neddylation process, but inevitably, it also encountered deficiencies like mutagenic resistance. Excitingly, researchers found that the UBC12-knockdown was capable of rescuing MLN4924-resistant H1299 cells, offering a great insight to conquer the MLN4924 resistance in lung cancer [34].

On the other hand, MLN4924 was also found to have effects like inhibiting the activity of lung cancer metastasis in recent years. Through treating both lung cancer cells and xenograft models with MLN4924 and utilizing a real-time whole-mouse imaging system to monitor the fluorescent stained Lewis lung carcinoma (LLC) cells protein expression site and metastatic process, it was discovered that MLN4924 affected the metastatic colonies’ intravascular survival, extravasation and formation, due to an inhibition of molecules like MMP2, MMP9 and vimentin together with an early-stage disruption of the actin cytoskeleton. It not only impaired the cell-invasive potential but also led to a series of results like apoptosis, cell cycle arrest, and DNA damage response. In the clinic, the expression levels of MMP2 and MMP9 were also upregulated in lung cancer patients with worse overall survival [51].

Moreover, the combination of MLN4924 and other compounds is also a promising approach in lung cancer. A study focused on a homologous recombination repair factor, BRCA1, which also served as a downstream part of the NSCLC neddylation process and PARP inhibitor targets, and treated NSCLC cells with MLN4924 to stepwise investigate whether the BRCA1 complex was affected. Then, results showed that MLN4924 could hamper the BRCA1 complex components’ recruitment to DNA damage sites to affect lung cancer progression. However, like BRCA1, it also indicated that MLN4924 was able to affect other PARP inhibitors to suppress the NSCLC growth. Then, a cotreatment of MLN4924 and PARP inhibitor, Olaparib, was performed in NSCLC cells, and the clinical lung cancer patient’s data was collected for a Kaplan-Meier survival analysis, which exhibited a better inhibition of the NSCLC cell growth. Also, there was a high pertinence between worse overall survival and the overexpression of NEDD8, BRCA1, and PARPs, confirming their previous hypothesis that MLN4924 was able to synergize with a PARP inhibitor for NSCLC treatment [52]. Recently, a phase II trial evaluated the efficacy of MLN4924 combined with docetaxel in patients with refractory stage IV NSCLC. The overall response rate was 22%, while the median progression-free survival was 4.1 months, and the median overall survival was 13.2 months. The data imply that the use of docetaxel alongside MLN4924 is active in treating patients with relapsed NSCLC [66].

#### 3.1.2 Candesartan Cilexetil

Previous studies had elucidated an overactivation of the neddylation pathway in various lung cancer progression and highlighted a significant therapeutic value of targeting the neddylation process as a clinical strategy [33]. Following it, in 2020, they screened 1331 approved drugs to test whether they were able to inhibit the cullin 1 neddylation through an improved enzyme-based assay. Then, their sight was locked on an anti-hypertensive agent, candesartan cilexetil (CDC), which exhibited a leading bioactivity via both *in vitro* and *in vivo* experiments, like apoptosis detection and tumor growth assays in A549 cells and a mouse model, CDC was observed to have considerable anti-cancer bioactivity. Further mechanism research revealed that it was mainly serving as a neddylation inhibitor that inhibited the cullin neddylation by competing with ATP to achieve the loss of NEDD8-activating enzyme. Most importantly, the effect of CDC was proven to be a higher execution priority than the representative NAE-inhibitor, M22, and mitoxantrone [54].

Besides, based on the CDC, another study designed 42 benzimidazole derivatives and synthesized them for a series of experimental verifications in 2021. With an IC_50_ almost three times lower than CDC (5.51 μM/16.43 μM), the benzimidazole-derived 35 showed an optimal anti-cancer effect among all 42 derivatives than CDC in lung cancer. Moreover, it could work as a promising neddylation inhibitor owing to its better targeting selectivity, which was proved in A549 cells and a docking model [55].

#### 3.1.3 HA-1141

In 2021, through the virtual screening and structural modifications, an MLN4924-like small molecule compound, HA-1141, was also found capable enough to directly bind to the NAE and inhibit cullin 1-5 neddylation at a well-tolerated dose in lung cancer. Besides, the HA-1141 effectively suppressed both the lung cancer cell proliferation *in vitro* and tumor growth in xenograft models. But it is worth noting that it showed a dual effect, as it could induce lung cancer cell autophagy through triggering the non-canonical endoplasmic reticulum (ER) stress and protein kinase R (PKR)-mediated terminal ISR. Overall, the relevance between HA-1141 and the cullin neddylation needs further research and exploration [56].

#### 3.1.4 HA-9104

In 2022, through the structure-based virtual screen and chemical optimization, Xu’s group first discovered a small molecule, HA-9104, which exerted a selectivity for targeting the UBE2F to inhibit the cullin-5 neddylation. As the CRL5-inactivation and NOXA-accumulation caused by the blockade of the cullin-5 neddylation, the HA-9104 was able to trigger apoptosis and suppress the lung cancer cell proliferation and tumor growth. In addition, the HA-9104 was also found to arrest the G2/M phase of cancer cells and cause DNA damage, which might be related to its sensitization effect on chemotherapy both *in vivo* and *in vitro*, showing a significant therapeutic potential for clinical lung cancer treatment [57].

#### 3.1.5 WS-384

The compound WS-384 was shown to selectively and actively inhibit the interaction between lysine-specific demethylase 1 (LSD1) and DCN1-UBC12 [58]. Then, WS-384 may trigger cell cycle arrest and apoptosis, with a marked reduction in tumor weight and volume in A549 xenograft mice. The underlying mechanism was that WS-384 promoted expression of p21 by blocking cullin 1 neddylation and diminishing H3K4 demethylation at the CDKN1A promoter.

### 3.2 Anti-Neddylation Effect and Mechanism of Natural Compounds from Herb Extract

Chinese medicine gradually exerted its importance in lung cancer treatment [67–69]. It is worth concentrating on the potential drug candidates with the ability to modulate neddylation in lung cancer.

#### 3.2.1 Piperidinyl Ureas and It’s Related Analogue

A study had discovered and elucidated a type of piperidinyl urea that was naturally inspired by piperine [70], exerting a regulatory effect on cullins with defective cullin neddylation 1 (DCN1)-dependent neddylation. In 2018, they reported an improved Structure-enabled optimization that not only significantly increased its biochemical potency to 100-fold, but also promoted the drug solubility and permeability to some extent. The most exciting thing was, the optimized compound exhibited a potential to hamper the DCN1-UBE2M interaction in a TR-FRET binding assay in lung squamous carcinoma cells, indicating its impact on neddylation. Then, in a series of other validations, like pulse-chase NEDD8 transfer assay, it was also proved to inhibit cullin neddylation due to a binding to DCN1 that would later reduce the steady-state levels of neddylated CUL1 and CUL3 [59]. Following it, their next research found an analogue from a series of the piperidinyl ureas, NAcM-OPT, which was potent in inhibiting the DCN1-UBE2M interaction, and it was an orally bioactive drug. Later, the NAcM-OPT showed its therapeutic value as a neddylation-targeted drug, and it was confirmed to inhibit the DCN1-mediated cullin neddylation together with affecting the NEDD8/CUL pathway, through a series of biochemical assays in lung squamous carcinoma cells with DCN1 amplification.

#### 3.2.2 Ethoxy-Substituted Phylloquinone (ESP)

In 2022, a study evaluated ESP, a compound extracted from spinach, through experiments like MTT and clonogenic assays for an examination of the anti-proliferation effect of ESP on both lung cancer cells (A549) and normal lung cells (WI38). It significantly but selectively hampered the A549 cells’ proliferation and led to their demise. Consistent with the in-silico analysis result, which indicated ESP owing features of a positive drug. Then, cell cycle analysis was also performed in A549 cells that were treated with ESP, showing an arrest in the G2-M phase. Mechanistically, through the SWATH-MS-based proteomic analysis, the anti-cancer effect of ESP was correlated with a decrease in neddylation pathway-related protein expression, along with an upregulation of STAT1 and NDRG 1 levels, and the EGFR signaling being affected [60].

#### 3.2.3 Arctigenin

As the traditional Chinese medicine is emerging exhibiting anticancer bioactivity, a study explored a natural extract database and first discovered the arctigenin, a main constituent of burdock, which had a suppression effect on various cancer malignant phenotypes due to its inhibition on UBC12, in 2022. Moreover, the arctigenin mainly hampered the activity of the UBC12 enzyme to affect the cullin neddylation and downstream pathways. Through proteomics analysis, the expression levels of cullin downstream substrates, like the tumor suppressor PDCD4, were also found an increase when cancer cells were treated with arctigenin [61].

#### 3.2.4 Gossypol

Through screening 17,000 compounds and conducting an α-Screen-Based high-throughput screen assay, a natural compound extracted from cotton seed, gossypol, was identified, showing an inhibition on cullin neddylation. Researchers elucidated that a direct binding of gossypol to the SAG-CUL5/RBX1-CUL1 complex, which mainly depends on the CUL5-H572, was the reason why this effect was achieved. In addition, when cullin neddylation was blocked by gossypol, a selective accumulation of CUL5 and CUL1 substrates, myeloid cell leukemia 1 (MCL1) or NOXA (PMAIP1) was observed in diverse cancer cell lines, including lung cancer. However, a synergistic effect between the gossypol and a specific MCL1-inhibitor was also confirmed, and it would suppress the cancer cell growth in the end [62].

#### 3.2.5 Norcantharidin

To search for a potent drug that was capable of saving various types of cancer radio-resistance, combining with their previous study, which revealed a protein CDC6 that could serve as a crucial molecule for overcoming radio-resistance, Deng’s group studied Norcantharidin (NCTD), a demethylated form of cantharidin that was extracted from the Chinese blister beetle. In this study, they confirmed that it was the neddylation-dependent pathways that the NCTD mediated to regulate CDC6 protein degradation, instead of normal Huwe1, Cyclin F, and APC/C related ubiquitin-proteasome pathways, emphasizing the therapeutic value for targeting neddylation. Furthermore, they explored which one of the cullin substrates (CUL1, 2, 3, 4A, 4B, and 5) was the key factor. The results from siRNA screening indicated that only cullin 1 was able to bind to CDC6, and NCTD exerted its effect by promoting this binding process, therefore, accelerating CDC protein degradation. This study also indicated that NCTD could combine with clinical radiotherapy for a better curative effect [63].

## 4 Conclusions and Perspectives

The protein precursors are not biologically active, and they will go through a final step of protein biochemical synthesis and PTMs to become mature proteins with different biological activities. As a covalent processing event, PTMs mainly exist in proteins like histones, secretory proteins, and membrane proteins, affecting them by adding various modifying groups to the amino acids [71,72]. Moreover, depending on the type of modifying groups, PTMs are classified into methylation, phosphorylation, SUMOylation, and ubiquitylation etc. [73], participating in a variety of biological processes such as regulation of the cell cycle, gene expression, and signal transduction [74]. In recent years, studies have revealed that PTMs are relevant to cancer progression as a crucial regulator [75,76], and neddylation, as a new type of PTM related to ubiquitylation, also shows great targeted value in cancer [77], especially with most articles focusing on lung cancer. Both ubiquitination and neddylation employ E1-E2-E3 enzyme cascades, but their substrates and biological outcomes differ. In lung cancer, ubiquitination has long been recognized as a fundamental driver of tumor progression, epithelial–mesenchymal transition, and drug resistance. Key ubiquitin E3 ligases, such as mouse double minute 2 (MDM2), CBL, and F-box and WD repeat domain containing 7 (FBXW7), as well as deubiquitinases (DUBs) like USP7 and USP9X, regulate oncogenic pathways, including p53 [78], EGFR, and PI3K/AKT [79]. Consequently, multiple ubiquitin–proteasome system (UPS) inhibitors have been developed—most notably proteasome inhibitors (e.g., bortezomib, carfilzomib) and DUB inhibitors targeting the 19S regulatory proteasome subunit (e.g., VLX1570) [80]. Despite their promise, these agents exhibit poor tumor selectivity, dose-limiting toxicity, and limited penetration into solid tumors, which has restricted their clinical success in lung cancer compared to hematologic malignancies. Moreover, the ubiquitination system is large and complex, which makes it difficult to develop drugs that directly target the core enzymes of ubiquitination due to their excessive toxicity. In contrast, the neddylation pathway is more streamlined. Its primary function is to activate CRLs by modifying Cullin proteins, thereby governing the ubiquitination and degradation of key proteins controlling core cellular processes. So, targeting its upstream NAE is an exceptionally attractive strategy. MLN4924 not only impaired the cell-invasive potential but also led to apoptosis, cell cycle arrest, and DNA damage response. Consequently, it is worthwhile to study the role of neddylation in cancer as a potential target [81,82], especially for lung cancer [57].

Although targeting neddylation could affect lung cancer metastasis through regulating various immune cells infiltration and overcoming drug-resistance of both lung cancer chemotherapy and targeted therapy, we have to mention that there is still a lot left to be explored in this field, like whether the lung cancer immune therapy-related resistance could also be saved by it. Also, there was a lack of clinical trial data in this field, and the neddylation hadn’t been explored yet on whether it could be combined with novel nano-targeted therapy to exert a better effect. Besides, when we searched related references on the therapeutic potential of neddylation, we found that other PTMs, like ubiquitination, could be affected by an interplay of the neddylation and SUMOylation complex [77], and there was also an interesting clue on targeting neddylation for other lung diseases, like acute lung injury (ALI) treatment [83], or the relationship between neddylation and the non-coding RNA [84], remaining for further exploration.

Taken together, this review pointed to neddylation as a potential therapeutic target in lung cancer treatment. Here, we not only mentioned the biological process of neddylation, highlighting its value in cancer treatment, especially lung cancer, but also summarized related studies on neddylation and lung cancer in recent years, explaining their association and mechanism in depth. Also, we highlighted that the neddylation played an essential role in lung cancer metastasis and drug-resistance, and concluded a series of potential drugs that exerted significant therapeutic value in lung cancer treatment via targeting neddylation.

## Data Availability

Not applicable.
